# Japanese experience of hydrogen sulfide: the suicide craze in 2008

**DOI:** 10.1186/1745-6673-5-28

**Published:** 2010-09-29

**Authors:** Daiichi Morii, Yasusuke Miyagatani, Naohisa Nakamae, Masaki Murao, Kiyomi Taniyama

**Affiliations:** 1 Department of Intensive Care Medicine, National Hospital Organization Kure Medical Center, Kure, 737-0023, Japan; 2 Division of Infection Control and Prevention, Osaka University Hospital, Suita, 565-0871, Japan; 3 Institute for Clinical Research, National Hospital Organization Kure Medical Center, Kure, 737-0023

## Abstract

Most of hydrogen sulfide poisoning has been reported as industrial accidents in Japan. However, since January 2008, a burgeoning of suicide attempts using homemade hydrogen sulfide gas has become evident. By April 2008, the fad escalated into a chain reaction nationwide. Mortality of the poisoning was very high. There were 220 cases of attempted gas suicides during the period of March 27 to June 15, killing 208. An introduction of new method of making the gas, transmitted through message boards on the internet, was blamed for this "outbreak". The new method entailed mixing bath additive and toilet detergent. The National Police Agency instructed internet providers to remove information that could be harmful. Of the victims of the fad in 2008, several cases were serious enough that family members were involved and died. Paramedics and caregivers were also injured secondarily by the gas. This fad has rapidly spread by internet communication, and can happen anywhere in the world.

## Overview

**Figure 1:**
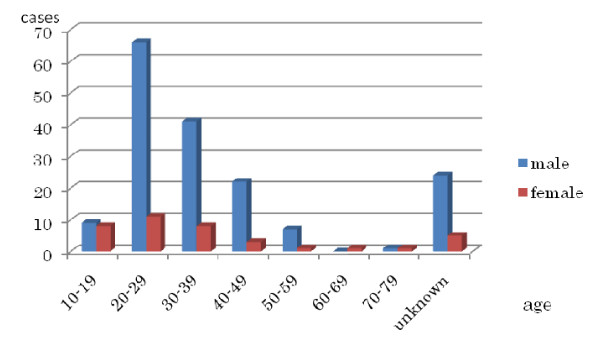
**220 cases during the period from March 28 to June 15, 2008**.

Hydrogen sulfide poisoning has been a relatively uncommon intoxication, with only a few cases a year being reported in Japan. Most incidents occurred in circumstances of volcano climbing, pharmaceutical product treatments, and man-hole cleaning[1]. Hence, this poisoning has been categorized as being associated with industrial accidents. However, since January 2008, there has been a burgeoning of suicide attempts using homemade hydrogen sulfide gas. By April 2008, the fad escalated into a chain reaction, and cases of H_2_S poisoning made headlines almost everyday, nationwide. The Japanese Cabinet Office reported 220 cases of attempted gas suicides during the period from March 27 to June 15, killing 208, a very high mortality rate (Figure 1). An introduction of new methods of making the gas, transmitted through message boards on the internet, was blamed for this "outbreak." The new method entailed mixing bath additive and toilet detergent. The main component of the bath additive is lime sulfur, and toilet detergent acts as an oxidant to produce H_2_S gas. In Japan, the custom of bathing, especially in hot springs (*onsen*), is quite common. As a result, people want to enjoy it in their own homes by using bath additive. These two materials are thus easily available in Japan, and also obtainable through the internet. Given these circumstances, the National Police Agency instructed internet providers to remove information that could be harmful, and MUTOHAP (the most frequently 'featured' brand of bath additives in the method) was forced to suspend its production. A few cases of swallowing MUTOHAP itself had already been reported as a means of suicide. If the sulfur in MUTOHAP were mixed with gastric acid in the stomach, a H_2_S gas-evolving reaction would occur and cause poisoning. When sulfur is mixed with a potent oxidant such as toilet detergent, an even greater quantity of H_2_S gas evolves than it would with gastric acid. In most of the cases, victims lose consciousness with a single intake of breath, and die immediately. This has been referred to as *knock down *and was introduced as a painless way to kill oneself.

This new method was first reported in 2007. Because of the burst of gas production in the reaction, it may involve passersby and rescue personnel, not just the person attempting suicide. Of the victims of the fad in 2008, several cases were serious enough that family members trying to rescue their sons or daughters were directly affected and died. In cases where the suicide attempt occurred in a hotel, guests were evacuated[2]. Because of its high water solubility, evaporated gas from the wet clothes of patients can cause secondary poisoning to paramedics and caregivers, too.

## Profile of hydrogen sulfide

Hydrogen sulfide is a colorless, hydrosoluble and toxic gas with a "rotten egg" smell. This gas is also flammable and can be volatile. It is pungent, often described as "rotten egg", even at concentrations as low as 0.05 ppm. At higher levels of exposure, a sweet odor can be sensed. Above 100 ppm, its warning odor is said to be lost, because of olfactory nerve paralysis (Table 1). The Japanese Society for Occupational Health sets 10 ppm as the maximum allowable concentration. Its gas specific gravity is 1.188 (comparable to air at 125°C and 1 atmosphere), meaning it is heavier than air. This is one reason why this gas is often associated with accidents in the sewer and mining industry. The gas is not only soluble in water, but also in petroleum.

**Table 1:** Effects of H_2_S at various concentrations

Concentration of H_2_S, ppm	Symptoms of exposure
0.05	Pungent smell mimicking "rotten egg"

0.1	Anosmia

50-150	Becoming paralyzed in a few minutes

250	Photophobia, lacrimation, rhinorrhea, cyanosis, pulmonary edema

250-500	Headache, nausea, vomiting, diarrhea, dizziness, palpitation, tachycardia, hypotension, muscle fasciculation, muscle weakness, apnea, disorientation, coma

500-750	Respiratory arrest within 30 to 60 min

750-1000	Collapsing momentarily or knocked down

>1000	Dying immediately within a breath

H_2_S inhibits enzymes in mitochondria by binding with Fe^3+ ^of cytochrome oxidase. This reaction blocks cellular respiration, and interferes with oxygen utilization at the cellular level. Cyanogen compounds act the same way, and the toxicity is similar. Treatment for H_2_S poisoning is similar to that for cyanogen compounds, as described below.

## Specific treatment

Nitrite salt may be efficacious. Nitrite salt oxidizes the Fe^2+ ^of hemoglobin (Hb) to Fe^3+^, deriving Met-Hb, which competes with the Fe^3+ ^of cytochrome oxidase and protects it from oxidization by sulfide. This mechanism is expected to ameliorate cellular anoxic conditions (Table 2).

**Table 2:** Treatment of H_2_S gas poisoning

Amyl nitrite	#. If spontaneous breathing remains, encourage amyl nitrite inhalation from the nasal airway tract.
	#. Until sodium nitrite is ready, repeat inhalation every 2 to 3 min.
Sodium nitrite	#. Dissolve 0.6 g sodium nitrite to 20 ml of distilled water for injection to make a 3% solution.
	#. Intravenously administer 10 ml (for child, 0.12-0.33 ml/kg) of the 3% sodium nitrite solution over 20 min or longer.
	#. Sodium nitrite is not on the market as a medicine, therefore, it requires preparation in each hospital using reagent sodium nitrite.

	#. Sodium thiosulfate is not efficacious, though it is used to treat cyanogen poisoning. (sodium thiosulfate does not have any negative effect for treatment of H_2_S poisoning.)

The efficacy and administration method of this drug have been discussed in some Japanese language articles. Here is a brief review of those findings[3]. The level of Met-Hb should be monitored when nitrite salt is used as a treatment for H_2_S poisoning. Although some experts say that the target Met-Hb level is approximately 30%, it seems feasible to keep the Met-Hb level under 25% with a concern of hypoxemia from methemoglobinemia. One anecdotal report described a case in which the patient was successfully saved with a maximum Met-Hb level as low as 14%. Although early administration of this treatment is desirable, there have been cases of both mortality and survival even after patients had entered a state of shock. Another anecdotal study reports that a patient survived without converting hemoglobin to methemoglobin by nitrite salt. However, the severity of those reported cases is assumed to vary, and the method of drug administration is not well established. There is insufficient data to support the widespread use of nitrite salt for H_2_S poisoning.

## Special concern for secondary disasters

Stirring bath additive and toilet detergent produces a great quantity of lethal gas, more than what is required for an individual suicide from H_2_S. Therefore, this can be deleterious for neighbors and rescuers. In the unfortunate fad of 2008, several families of people who attempted suicide became victims themselves. Paramedics and caregivers were also reported to have become injured secondarily. The Tokyo Fire Department alerted family members, neighbors, and hotel staff not to enter any rooms where H_2_S was suspected to have been made. Closed rooms or cars proved to be extremely dangerous to enter in an attempt to save loved ones or customers before paramedics arrived.

For paramedics and caregivers, management of a C disaster based on the NBC (Nuclear, Biological and Chemical) disaster is sometimes necessary. After a patient is evacuated, first-step procedures or treatments should be performed in an airy space. Undressing, dry decontamination, is undoubtedly necessary, and if discolored skin is evident, water decontamination such as showering should also be considered. Because H_2_S gas is detected in patient expiration, mouth-to-mouth resuscitation is not indicated. An ambulance is a small, enclosed space, so exhaled H_2_S gas from a patient can potentially cause poisoning of paramedics. When transferring a patient with H_2_S poisoning, all windows should be opened and the vehicle should be well ventilated. Accurate decontamination in the field and in-car ventilation are the most important things to keep paramedics safe from secondary injury. In the same way, caregivers should treat and decontaminate patients outside of the hospital, behind partitions, for example. However, in most of the cases of H_2_S suicide, the victim is the only person to treat. Considering the time it takes to set up a partition, it is not clear how far we should proceed with this method.

In conclusion, H_2_S gas suicide attempts are of an extremely high mortality rate. The gas can also injure family, paramedics and caregivers. More research is needed into the potential dangers to first responders before hospitals and other agencies can make comprehensive plans about how to deal with victims. This fad spread rapidly by internet communication, and can happen anywhere in the world with chemicals readily available for purchase online.

## Competing interests

The authors declare that they have no competing interests.

## Authors' contributions

All authors read and approved the final manuscript.
